# Ontology-Based Medication Named Entity Recognition Using Pretrained Transformer Models From a Thai Hospital: Model Fine-Tuning and Validation Study

**DOI:** 10.2196/82685

**Published:** 2026-03-20

**Authors:** Natthanaphop Isaradech, Wachiranun Sirikul, Stefan Schulz, Markus Kreuzthaler

**Affiliations:** 1 Environmental and Occupational Medicine Excellence Center, Faculty of Medicine, Chiang Mai University, Chiang Mai, Thailand; 2 Department of Community Medicine, Faculty of Medicine, Chiang Mai University, Inthawarorot Road, Si Phum, Mueang, Chiang Mai, 50200, Thailand, 66 53935472; 3 Department of Biomedical Informatics and Clinical Epidemiology, Faculty of Medicine, Chiang Mai University, Chiang Mai, Thailand; 4 Center of Data Analytics and Knowledge Synthesis for Health Care, Chiang Mai University, Chiang Mai, Thailand; 5 Institute for Medical Informatics, Statistics and Documentation, Medical University of Graz, Graz, Austria

**Keywords:** knowledge representation, ontology, named entity recognition, information extraction, deep learning, annotations, electronic medical prescriptions, natural language processing

## Abstract

**Background:**

Extracting accurate medication information from Thai hospital records presents challenges due to the narrative style of medical notes, which often combine Thai and English terminology. Named entity recognition (NER) serves as the foundational step for advanced clinical information extraction (IE) tasks, including medical concept normalization and relation extraction. This study aimed to establish a robust NER framework to address these difficulties by leveraging ontology-based annotation and pretrained transformer models.

**Objective:**

The primary objective of this study was to evaluate the performance of 5 fine-tuned pretrained transformer models—BioClinicalBERT, ClinicalBERT, PubMedBERT, MultilingualBERT, and ThaiBERT—based on Bidirectional Encoder Representations from Transformers (BERT) in extracting structured medication information from unstructured Thai hospital discharge summaries.

**Methods:**

Ninety discharge summaries were collected from Maharaj Nakhon Chiang Mai Hospital. These documents were annotated by physicians following the annotation guidelines based on international standards, including Systematized Nomenclature of Medicine-Clinical Terms (SNOMED-CT) and Health Level Seven Fast Healthcare Interoperability Resources (HL7 FHIR). The dataset was divided into fine-tuning (70 records, 78%, 2030 annotated spans), validation (10 records, 11%, 277 annotated spans), and testing sets (10 records, 11%, 358 annotated spans). The 5 transformer models were fine-tuned and evaluated using this annotated data to recognize and classify key medication entities (substance, route of administration, unit of measure, time patterns, and unit of presentation).

**Results:**

We found that all models had good NER performance metrics in both the validation and test datasets. Regarding test performance, ClinicalBERT achieved the highest exact *F*_1_-score at 0.973, compared with 0.968 for BioClinicalBERT, 0.925 for PubMedBERT, 0.931 for MultilingualBERT, and 0.969 for ThaiBERT. All models showed strength in accurately identifying “Substance” and “Dosage” entities, whereas “Unit of Measure” proved to be the most challenging entity type due to implicit information in the source text for all models.

**Conclusions:**

The findings suggest that ontology-based medication IE using transformer-based models holds promise for enhancing data standardization and interoperability within the Thai health care system. Future work will need to leverage the granular annotations preserved in the dataset to develop medical concept normalization and relation extraction models to complete the medical IE system.

## Introduction

### Background

Over the last few decades, hospital information systems have continuously evolved from paper-based to electronic systems [1], with the promise of more efficient data collection and retrieval, as well as reducing the likelihood of errors [2,3]. Electronic health records (EHRs), hosted by hospital information systems, were initially implemented to replace paper charts but are increasingly regarded as valuable resources for health data analysis. While standards orientation and interoperability on an ontological basis [4] have been increasingly advocated, much of the EHR content is still written in a narrative style, containing crucial details about the patient, such as chief complaints, present illness, past medical history, past procedures, physical examinations, and even medication, as free text. Free text ideally supports quick human communication but poses significant challenges to content retrieval [5-7]. Only small portions of EHR content are currently structured using semantically explicit identifiers, such as *ICD-10* codes for diseases. Additionally, medication prescriptions are still mostly written in free text, despite the high relevance of medication errors to patient safety.

In Thailand, the writing style of narrative drug administration statements in discharge summaries is particularly unique due to its blend of Thai and English vocabulary, tailored for Thai physicians. They commonly mix brand names with ingredient names and tend to omit units and dosage forms such as tablets, suspensions, and eye drops whenever this does not lead to ambiguous interpretations. Style and format vary depending on the physicians’ preferences and local communication norms. For example, in “fluimucil (100) 1 ซอง ผสมน้ำ po bid,” “fluimucil” is the trademark name with “N-acetylcysteine” as an active ingredient, “100” indicates strength (with “mg” omitted because it is known that other units are not used for this medication), “1” refers to the dose quantity for this drug, and “ซอง” is a Thai word that means “sachet.” “ผสมน้ำ” is an instruction in Thai meaning “mix with water” in English. “po” stands for “per os” (by mouth), and “pc” means “postprandial” (after meals) and “bid” is an abbreviation meaning “two times a day.” This flexible and overly compact form poses challenges for the correct and fine-grained extraction of drug information from narrative content in EHRs.

### Extracting Medication Information

Many studies have described natural language processing (NLP)-based information extraction (IE) for secondary use to improve health care research, health interoperability, hospital data analysis, and more [8]. IE from biomedical text includes 2 important steps: named entity recognition (NER) together with medical concept normalization (MCN) on the one hand, and relation extraction (RE) on the other hand. NER identifies spans of text and classifies them into broad semantic categories according to what they denote (eg, drugs, symptoms, procedures, and medication strength). MCN refers to mapping these spans to standardized terms as provided by SNOMED-CT (systematized nomenclature of medicine-clinical terms), *ICD-10*, and HL7. Some vocabulary is grounded in ontologies, that is, formal descriptions of their referents, in our context, particularly SNOMED-CT. However, effective normalization (MCN) relies heavily on the accuracy of the preceding step: identifying exactly where an entity begins and ends. If the NER model fails to correctly separate a “Substance” from its adjacent “Dosage Instruction,” any subsequent attempt to map these spans to standard ontology codes will result in errors or misclassifications. Hence, robust NER is a necessary structural prerequisite for semantic interoperability.

RE involves identifying and classifying binary relations between text spans (also known as entities) as delineated by NER. Typical relations include the *location* relation between a disease and a body part, or the *value* relation between a laboratory observable and a number. In medication administration statements, typical relations connect a product name to the active ingredient(s), an ingredient to a dose quantity, and the whole medication statement to a temporal value. Some relations can also be mapped to slots in HL7-FHIR (Health Level Seven Fast Healthcare Interoperability Resources) resources.

IE in the clinical domain has evolved through several methods. Early systems, such as MetaMap [9], KnowledgeMap [10], and cTAKES [11], relied on rule-based pattern matching and terminology lookup. These were complemented by supervised machine learning approaches—particularly conditional random fields (CRFs) and support vector machines—which demonstrated strong performance in benchmark challenges such as i2b2 [12]. More recently, deep learning (DL) methods, including recurrent neural networks and transformers, have shown improved ability to capture contextual semantics in complex clinical narratives. Peterson and Liu [13] showed how DL, in particular convolutional neural network and bidirectional long short-term memory models, fared well in assigning SNOMED-CT codes to text expressions and relations. Mahendran and McInnes [14] showed that an approach based on Bidirectional Encoder Representations from Transformers (BERT)-based models outperformed other models overall and obtained state-of-the-art performance in adverse drug event extraction with an *F*_1_-score of 94%. Hristov et al [15] proposed a multistep approach utilizing clustering, fine-tuned BioBERT classification, and mapping to predict SNOMED-CT codes for clinical texts, showing high accuracy and reliability. While “Generative artificial intelligence (AI)” (large language models) has recently gained prominence for zero-shot and few-shot tasks, we distinguish these from the encoder-based BERT models (Transformers) utilized in this study. The choice to prioritize fine-tuned BERT models over generative AI was driven by the specific requirements of clinical data extraction. In our recent study, we investigated the use of ChatGPT to perform NER tasks on the same Thai medication prescription data used in this research [16]. While that study yielded a promising average strict precision of 0.84, the model exhibited significant instability, evidenced by a poor recall of 0.66 for medication instructions. This limitation raises major concerns, as misclassification by generative AI could result in missing critical prescription details, such as dose quantity, timing, and route. Consequently, fine-tuned BERT-based models remain the superior choice for high-recall clinical extraction.

Despite these global advancements in model architecture, we found a significant lack of application of model-based NER and gold-standard datasets suitable for hospital settings in Thailand, largely due to the unique style of drug administration expressions exemplified above. Therefore, we created an annotation guideline to standardize and enrich our drug administration dataset, making it trainable and evaluable for robust BERT architectures.

### The Ontology of Medication Statements

The ontological analysis of a domain requires identifying, characterizing, and classifying the types of entities and how they are related. It helps clarify the basic assumptions on which knowledge representation artifacts are built. Main categories of being and foundational relations are provided by so-called foundational ontologies [17]. We here refer to the Basic Foundational Ontology (BFO), which has had a strong influence on domain ontologies in biology and medicine. In the domain of medication, we have the medicinal product at the center. It is a material artifact prepared for entering contact with a biological organism to produce some effect by interacting with the organism’s metabolism. The medicinal product is characterized by being composed of one or more ingredients, ie, amounts of substances. Substances are collections of molecular units of a certain type. For example, sodium chloride is a substance consisting of equal amounts of sodium and chloride ions; saline is a substance containing additional water molecules as a solvent. Every medicinal product, which is, in contrast to substances, of a discrete nature (it can be portioned and counted), has one or more substances as parts. We here only consider the active substances. Each constituting substance has a defined quantity, which is characterized by a number and a unit of measurement, which is a dimension or physical quantity (milligrams, millimoles, milliliters, etc), providing a reference based on which the exact number of molecular entities can be determined. Every medicinal product has a dose form, eg, tablet, cream, or solution for injection, which optimizes the way the product’s ingredients reach the target body structure. Dose forms can ontologically be described as qualities of the medicinal product. Dose form, active ingredients, and the amount of each ingredient characterize what SNOMED-CT names “clinical drug,” a class that can be further divided according to the branded drugs of different manufacturers. From a clinical point of view, these subdivisions are of minor interest.

However, the context in which a medicinal product is referred to is diverse. Not all of its mentions pertain to actual medication administration processes. Alternatively, mentions can refer to past administrations, possibly with potential adverse effects, but medication mentions can also occur in the context of prescriptions or recommendations. All of these are, ontologically, processual entities or, at the level of SNOMED-CT, procedures, but their impact on the real exposure of a patient to a pharmacologically active substance is different. Processual entities, in BFO, have a specified time and location. In contrast to medication administration events, prescriptions or recommendations are plans or intentions that might not necessarily be realized in the future. In the medication section of clinical summaries, it is not always clear whether the entries refer to actual medication administration events or mere recommendations.

To translate these foundational principles into a practical schema, we developed the “Medication Annotation Guideline,” primarily grounded in SNOMED-CT, which provides the semantic foundation for our entity types. Each NER label corresponds to a specific SNOMED-CT concept node using the standard format, Concept ID |Fully Specified Name|: for example, a text span annotated as Substance must conceptually descend from 105590001 |Substance|, and Route must descend from 284009009 |Route of administration value| (see Table 1 in Methods). For aspects beyond SNOMED-CT’s scope as a terminology—such as dose quantity values and dosage timing structures—we relied on HL7 FHIR as a complementary framework, adopting elements from its Medication Statement resource (see Table 2 in Methods). This provides annotators with precise, unambiguous inclusion criteria, improves inter-annotator consistency, and ensures that entities extracted by our NER models are aligned with international interoperability standards.

To conclude, IE is a multistage process required to fully represent unstructured clinical data. A complete IE pipeline typically involves 3 distinct tasks: NER to identify and classify text spans, MCN to map those spans to standardized terminology codes, and RE to link these entities together.

This study focuses on the NER task as it is the foundational stage of IE. We employed an ontology-based annotation strategy using SNOMED-CT primarily as it aligns with the clinical reasoning and terminology native to annotators, who are medical professionals, and this also guarantees the dataset’s extensibility: by strictly adhering to SNOMED-CT standards now, the same annotated corpus can be directly utilized for future MCN and RE tasks, bridging the gap between extracting text spans and achieving full semantic interoperability.

**Table 1.** Entity types relevant to the medication domain, including SNOMED CT top concepts and corresponding basic foundational ontology classes.

Acro	Code	SNOMED CT^a^ name	BFO^b^ class	Description	Examples
*Sub*	105590001	Substance	object aggregate	Drug names, active ingredients, medicinal compounds	Paracetamol, Metformin, Amoxicillin, ASA (aspirin)
*Uni*	767524001	Unit of measure	generically dependent continuant	Units for dose quantity measurement	tab (tablet), cap (capsule), amp (ampule), vial, sachet
*Tim*	272103003	Time patterns	occurrent	Dosing frequency, timing, or duration	1*1 (once daily), bid, prn, pc (after meals), hs (at bedtime)
*Rou*	284009009	Route of administration value	object	Route of administration - how the drug enters the body	po (per oral), IV, SC (subcutaneous), IM, topical
*Adm*	18629005	Administration of drug or medicament	process	The procedure/event of giving medication to patient	(*zero-width annotation - implicit, not tied to text span*)
*Pro*	763158003	Medicinal product	object	The manufactured drug product being administered	(*zero-width annotation - implicit, not tied to text span*)
*Pre*	732935002	Unit of presentation	quality	Physical dosage form of the medication	tab (tablet), cap (capsule), amp (ampule), vial, sachet
*Dec*	1119403002	Decimal	generically dependent continuant	Numerical values for dose strength or quantity	500, 10.5, 0.25, 100/10 (compound strength)

a SNOMED CT: Systematized Nomenclature of Medicine-Clinical Terms.

b BFO: basic foundational ontology.

**Table 2.** Predicate values, their domain and range restrictions, and their interpretation as Systematized Nomenclature of Medicine-Clinical Terms (SNOMED CT) and Fast Healthcare Interoperability Resources (FHIR) expressions.

Predicate	Domain/Range	Relational expression	BFO^a^ relation
doseForm	*Adm* → *Pre*	411116001 |Has manufactured dose form	bearer of
doseQuantity	*Adm* → *Dec*	FHIR:Dosage.doseAndRate.dose	bearer of
doseRoute	*Adm* → *Rou*	410675002 |Route of administration	located in
doseTiming	*Adm* → *Tim*	FHIR:Dosage.timing	has occurrent part
ingredient	*Pro* → *Sub*	127489000 |Has active ingredient	has continuant part
value	*Sub* → *Dec*	1142138002 |Has concentration strength numerator value 1142135004 |Has presentation strength numerator value	bearer of
unit	*Sub* → *Uni*	1142135004 |Has presentation strength numerator value 732945000 | Has presentation strength numerator unit	bearer of
usingSubstance	*Adm*_→_ *Pro/Sub*	363701004 |Direct substance	has participant

a BPO: basic foundational ontology.

In the following sections, we describe how these concepts guided our annotation process and demonstrate how the resulting dataset was utilized to enable transformer-based models to learn and extract structured medication information from unstructured Thai clinical documents.

## Methods

### Ethical Considerations

This study was approved by the Research Ethics Committee, Faculty of Medicine, Chiang Mai University, Thailand (approval number COM-256509305, Research-ID: 9305). Consent to participate was waived in accordance with the exemption for secondary data used exclusively for research purposes. There was no recruitment of or compensation provided to participants for this study. All discharge summaries were deidentified prior to annotation: patient identifiers (names, hospital numbers, dates of birth, addresses, and other personally identifiable information) were removed before the data were made available to annotators. Annotators only accessed the deidentified text. The deidentified dataset was stored on a secure institutional server with access restricted to authorized research team members.

### Study Objectives

This study aimed to first construct an ontology-based annotated corpus for Thai medication statements aligned with the international terminology standards, SNOMED-CT and FHIR. Second, we utilized this corpus to develop and validate 3 fine-tuned transformer models based on their performance in NER to extract specific medication entities—including substance, route of administration, unit of measure, time pattern, and unit of presentation—from Thai English code-switched clinical text.

### Data Acquisition and Ontological Interpretation

Ninety discharge summaries with narrative content from the Maharaj Nakhon Chiang Mai Hospital, spanning the years 2018 to 2022, were randomly collected. The documents were sourced mainly from the Department of Internal Medicine, as it is the department where medical vocabularies and medication prescriptions are the most diverse. Moreover, it is the department that employs a large rotation of attending physicians, resulting in a mix of heterogeneous writing styles within the dataset. Each discharge summary document contains the complete clinical narrative for a single, unique patient admission. The following exclusion criteria were applied: (1) records with incomplete or corrupted text fields; (2) cases resulting in death, which differ significantly in documentation style; and (3) discharge summaries containing zero medication entries. Each discharge summary document contains the complete clinical narrative for a single, unique patient admission. To balance learning capability with evaluation, we reviewed comparable clinical NLP studies and found that training set allocations typically range from 60% to 75% [18-20]. Accordingly, we adopted a similar distribution:

- Training Set (n=70, 78%): Used for fine-tuning.
- Validation Set (n=10, 11%): Used for hyperparameter tuning and model checkpoint validation.
- Test Set (n=10, 11%): Used for performance evaluation on unseen patients.

The dataset was annotated by 3 general physicians according to a Medication Annotation Guideline. The summaries are divided into 3 groups (33, 33, and 34 summaries). Each of the 3 annotators was assigned to annotate 2 groups, ensuring that each summary received 2 versions of annotations by different annotators.

In Thailand, discharge summaries frequently adhere to distinct patterns among hospitals and institutes. These summaries typically include a concise summary of the patient’s demographics, chief complaint, current illness, past history, physical examination, problem lists, and medication prescriptions for each hospital admission. In our dataset, we interpret medication statements as denoting real drug administration procedures, ie, we can assume as real that the patient received the mentioned products. Due to our knowledge of workflows and documentation practices, we take for granted that each medication statement denotes a real-world instance of SNOMED-CT code 18629005 |Administration of drug or medicament (procedure)|. As we can trivially assume, every instance of this concept refers to some medicinal product, as introduced above. In BFO, the products would be the participants of these administrative procedures. These procedures can be further specified in terms of the quantity of the unit over a period, the time patterns, and the route by which it enters the body. A time pattern, eg, 3 tablets a day at 7 AM, 2 PM, and 9 PM, corresponds to 3 subprocedures per day, characterized by a different time each. The route would be referred to as the anatomical location (mouth, skin, vein, etc) where each of the subprocedures takes place.

### Medication Annotation Guideline

#### Development of Annotation Guidelines

These ontological assumptions motivated the creation of our annotation guideline, which was developed based on the standards SNOMED-CT and FHIR. It had to synthesize the perspectives of foundational ontology, the clinical ontology SNOMED-CT, and the information model HL7-FHIR, inspired by the AIDAVA annotation guideline [21,22], from which annotation training materials were derived and which were used to resolve annotation controversies. We used INCEpTION v32.1 for text annotation [23]. The medication annotation tasks are explained in the following 2 steps:

#### Entity Annotation

SNOMED CT is used as the main annotation terminology. The annotation process followed several rules. The first rule was “Use the most fine-grained SNOMED-CT concept.” Entity annotations (ie, annotations of text spans) are performed using the most fine-grained SNOMED-CT concept possible, which represents the correct meaning of the surface term under investigation to avoid ambiguity and ensure a higher level of precision, since any necessary generalization can be applied later if required by the downstream task, rather than trying to refine a broad concept initially.

The second rule was “Zero-width annotations (not rooted in any text) are allowed only for 105590001 |Substance (substance)| and 18629005 |Administration of drug or medicament (procedure)|.” These 2 concepts are crucial for expressing the meaning of a medication expression but are often only implicitly referred to [22]. Despite being annotated with fine-grained SNOMED CT codes for the provision of entity types for NER and without interest in MCN in this study, we had to map them to a small set of 8 upper-level SNOMED-CT concepts, as shown in Table 1.

The third rule was “‘Clinical drug’ concepts are not allowed.” In narrative medication statements, many mentioned texts refer to branded or clinical drugs, which are not listed in SNOMED-CT, where the most detailed drug product concepts, fully defined by strengths, dose forms, and ingredients, are identified with the “Clinical drug” tag. This may cause ambiguity for annotators, for example, when choosing either 428789004 |Product containing precisely simvastatin 20 milligram/1 each conventional release orodispersible tablet (clinical drug) or 319997009 |Product containing precisely simvastatin 20 milligram/1 each conventional release oral tablet (clinical drug)|, as the difference between these 2 concepts is that one has “orodispersible tablet” and the other has “oral tablet,” which may not be explicitly distinguished in the written medication statement. To avoid confusion for annotators, we excluded the clinical drug concepts from our annotation vocabulary, all the more because their coverage is currently limited. In addition, the fact that the expressions relate to medication administration is mostly implicit. This is the reason why we added 18629005 |Administration of drug or medicament (procedure)| as a zero-width annotation, ie, an annotation to which no specific text span is related. The same is often needed for 763158003 |Medicinal product (product)| in cases where only the substance is referred to in the medication statement.

#### Relation Annotation

The relation annotation uses the predicates outlined in Table 2, taken from the AIDAVA annotation guide [21,22]. These predicates prioritize human understandability to facilitate the annotation process. Their semantic interpretation in terms of SNOMED CT and FHIR is listed in column 3, and their abstraction to BFO relations is provided in column 4.

Domain and range restrictions in Table 2 use the acronyms from Table 1. DoseForm relates the medicinal product to its dose form, for example, tablet, capsule, solution, etc. DoseQuantity relates drug administration to the count or amount of the drug given. If available in discrete portions, for example, tablets, it links to an integer; otherwise, it links to a number that quantifies an amount, for example, “20 mL/h.” DoseRoute indicates the path through which a substance enters the body (eg, oral route, intravenous infusion, or inhalation). DoseTiming links drug administration to dosing schedule concepts, for example, “every 8 hours”, or specific dates and times like “for 10 days before breakfast.” An ingredient relates a medicinal product to its active ingredient(s). Value relates an ingredient to the mass or concentration present in a dosage unit of the product, together with the unit of measurement. UsingSubstance describes the relationship between drug administration and the medicinal product or the substance (if no product is specified).

### Annotation Examples

**Figure 1.**
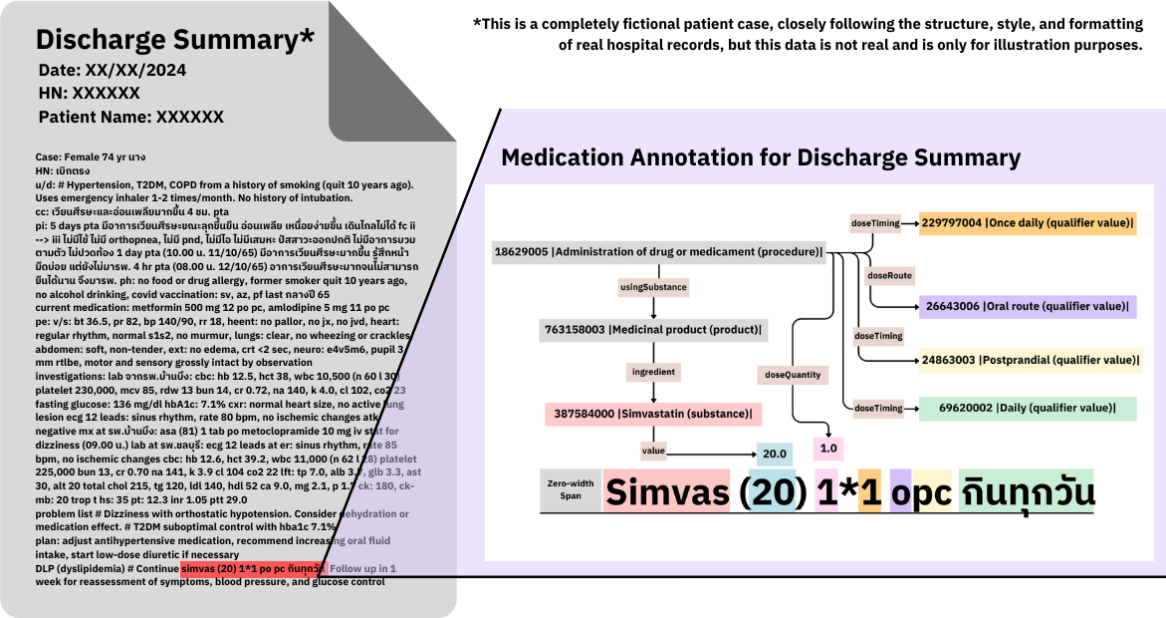
Example 1 of the medication administration annotation from the discharge summary note.

From the Figure 1 example, “simvas(20) 1*1 opc” means “taking Simvastatin 20 mg 0.5 tablet once daily,” The annotation starts with 18629005 |Administration of drug or medicament (procedure)| linked to 763158003 |Medicinal product (product)| by the usingSubstance predicate and links 763158003 |Medicinal product (product)| to all of its ingredients, in this case, 387584000 |Simvastatin (substance)|, using the ingredient predicate. Then we link the dosage of the substance, “20.0,” to 387584000 |Simvastatin (substance)| using the value predicate.

The first number “1” in Thai medication prescription style indicates doseQuantity, ie, without using abbreviations or ambiguous terms. We annotated “0.5” as a decimal and linked it to 18629005 |Administration of drug or medicament (procedure)| using doseQuantity. The second occurrence of “1,” as well as “pc,” indicates doseTiming, so we linked 229797004 |Once daily (qualifier value)| and 24863003 |Postprandial (qualifier value)| to 18629005 |Administration of drug or medicament (procedure)| using doseTiming. “o” indicates the route of administration, so we linked 18629005 |Administration of drug or medicament (procedure)| to 26643006 |Oral route (qualifier value)| using doseRoute. Finally, “กินทุกวัน” is “to be consumed daily” in Thai, so we annotated it with 69620002 |Daily (qualifier value)| linked to 18629005 |Administration of drug or medicament (procedure)| using doseTiming.

**Figure 2.**
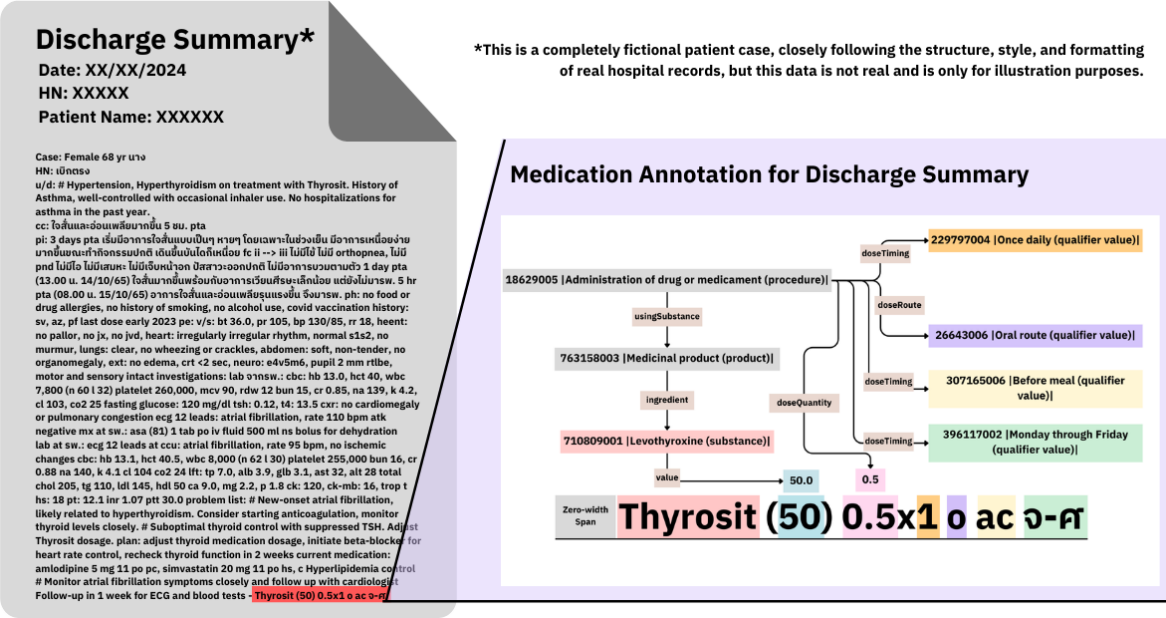
Example 2 of the medication administration annotation from the discharge summary note.

In Figure 2, “Thyrosit (50) 0.5x1 o ac จ-ศ,” the annotation begins with 18629005 |Administration of drug or medicament (procedure)| linked via usingSubstance to 763158003 |Medicinal product (product)|, which connects to 710809001 |Levothyroxine (substance)| through the ingredient predicate. The strength “50” is linked to the substance using value. The “0.5” indicates doseQuantity (half tablet), linked to the administration procedure. The expression “x1,” combined with the Thai abbreviation “จ-ศ” (จันทร์-ศุกร์, meaning Monday through Friday), creates multiple doseTiming relations: 229797004 |Once daily|, 307165006 |Before meal| (from ac), and 396117002 |Monday through Friday|. Finally, “o” indicates 26643006 |Oral route| via doseRoute.

### Gold Standard Dataset Evaluation

The agreement between 3 annotators at both the concept and relation levels was determined by Krippendorff α [24], a statistical measure that evaluates the level of agreement among raters while accounting for the possibility of agreement occurring by chance.

### Model Selection and Fine-Tuning

We chose the following pretrained transformer models for model fine-tuning: (1) BioClinicalBERT [25], (2) ClinicalBERT [26,27], (3) PubMedBERT [28], (4) multilingualBERT [29], and (5) ThaiBERT [30]. ThaiBERT and multilingualBERT were included as baseline models to evaluate how general-domain language models perform on this specific NER task compared to domain-specific models. Specifically, ThaiBERT was selected to assess the performance of a model pretrained primarily on native Thai text, while multilingualBERT was chosen to investigate how a multilingual model—capable of processing both English and Thai but lacking specific biomedical background—handles the code-switched clinical narratives.

The selection of models pretrained on English corpora (BioClinicalBERT, ClinicalBERT, and PubMedBERT) is guided by the writing nature of Thai clinical documentation, which is predominantly English-centric but frequently incorporates Thai text and grammatical structure. Although Thai characters will be treated as out-of-vocabulary tokens (unknown), we believe English-pretrained models can still perform well on our dataset because they were extensively trained on vast biomedical corpora, which directly align with the medication names and clinical terms that dominate Thai clinical documentation.

After each fine-tuning epoch, the model was evaluated on the validation set. The checkpoint achieving the highest validation *F*_1_ score was selected and subsequently evaluated for generalizability on the test set.

### Data Preprocessing and Tokenization

The extracted discharge summaries were composed of unstructured narratives. To prepare the text for analysis, we performed a cleaning step to filter out special characters, common stopwords, and non-Thai or non-English vocabulary.

Then, we transformed fine-grained concepts into entity types based on SNOMED-CT, as shown in Table 1. Each fine-grained code annotated by experts (eg, 387584000 |Simvastatin (substance)|) was traced through its parent hierarchy (IS-A relationships) in SNOMED-CT until it matched one of the 8 target upper-level concepts defined in Table 1. For example:

- 387584000 |Simvastatin| → IS-A → 105590001 |Substance| → Entity type: SUB
- 26643006 |Oral route| → IS-A → 284009009 |Route of administration value| → Entity type: ROU
- 229797004 |Once daily| → IS-A → 272103003 |Time patterns| → Entity type: TIM

18629005 |Administration of drug or medicament (procedure)| and 763158003 |Medicinal product (product)| were excluded from model development and validation because these are zero-width annotated concepts and, therefore, inferable from the ontology pattern.

Text processing was then performed using the specific subword tokenization strategies associated with each pretrained model. English pretrained models (BioClinicalBERT, ClinicalBERT, PubMedBERT), lacking a Thai vocabulary, map all Thai characters to (unknown). Consequently, they treat Thai text as delimiters and rely entirely on English medical terminology as semantic anchors. MultilingualBERT uses a multilingual vocabulary. It applies standard subword tokenization to English but often fragments Thai script into individual characters or subcharacter units due to the lack of dedicated Thai word segmentation. ThaiBERT uses a SentencePiece tokenizer trained on Thai Wikipedia. However, due to its Thai-dominant vocabulary, it lacks technical English terms and might fragment them into small, generic subwords.

### Model Evaluation

Precision (P), recall (R), and the microaveraged *F*_1_-score were used to assess model performance. The equations for these evaluation metrics are illustrated as follows:


$$Precision=\frac{TruePositive}{\left(TruePositive+FalsePositive\right)}$$

$$Recall=\frac{TruePositive}{\left(TruePositive+FalseNegative\right)}$$

$$F1-Score=\frac{2\times (Precision\times Recall)}{\left(Precision+Recall\right)}$$

In the assessment, we differentiated between exact match and partial match [19,31-33]. Exact Match (Strict) requires the predicted entity to match the ground truth exactly in both its boundary (start and end indices) and its entity label. Partial Match (Relaxed) counts a prediction as correct if the predicted span overlaps with the ground truth span (even partially) and the entity label is correct.

For example, consider a medication prescription, “Amlodipine (5), 1*1 po pc หลังอาหารเช้า,” where the ground truth for a Substance entity is marked as “Amlodipine.” If the model predicts “Amlodipine (5),” this would be classified as a failure under the exact match criterion because the character boundaries do not align perfectly, but it will be classified as correct under the partial match criterion because the core drug name (“Amlodipine”) is present, although the predicted span includes the dosage suffix “(5).”

### Statistical Analysis

Statistical comparison of model performance metrics, including both exact and partial recall, precision, and *F*_1_-score, was performed by assuming correct predictions as binomial proportions. A chi-square test for equality of proportions was initially applied to detect global differences in performance metrics among the 5 pretrained transformer models (BioClinicalBERT, ClinicalBERT, PubMedBERT, MultilingualBERT, and ThaiBERT). For the metrics that showed significant global differences, pairwise comparisons were performed using immediate 2-sample proportion tests (*prtesti* in STATA). The Bonferroni method was applied to correct for the 10 possible pairwise comparisons (5 models) per metric. Statistical significance was defined as a Bonferroni-corrected *P* value <.05. The statistical analysis was conducted using STATA version 17 (STATA Corp.).

## Results

The evaluation of the annotated data revealed that Krippendorff α coefficients among the 3 annotators were 0.817 for entity type annotations and 0.960 for relation annotations. Table 3 presents the distribution of token types across the fine-tuning, validation, and test datasets for the NER task. Table 4 presents the development and validation results for the 5 fine-tuned models. ClinicalBERT achieved the highest overall performance, reaching an *F*_1_-score of 0.974 for both exact and partial evaluations. Notably, ThaiBERT demonstrated strong competitive performance, securing the second-highest *F*_1_-score of 0.970, outperforming BioClinicalBERT (0.965). In contrast, MultilingualBERT and PubMedBERT yielded lower performance metrics, with *F*_1_-scores of 0.935 and 0.933, respectively. The high precision scores across all models (>0.990) indicate that false positives were rare, while the variations in recall suggest that ClinicalBERT and ThaiBERT performed the best in the NER task for our dataset.

**Table 3.** Number of annotated spans per category in fine-tuning, validation, and test data.

Dataset	Number of documents	Substance	Decimal	Route of administration	Time patterns	Unit of presentation	Unit of measure	Outside entity
Fine-tuning	70	441	742	307	469	55	16	35,358
Validation	10	58	103	44	62	8	2	4626
Test	10	75	128	62	83	6	4	4626

**Table 4.** Overall performance metrics for BioClinicalBERT, ClinicalBERT, PubMedBERT, MultilingualBERT, and ThaiBERT on the fine-tuning and validation datasets.

Metrics	BioClinicalBERT	ClinicalBERT	PubMedBERT	MultilingualBERT	ThaiBERT
Exact precision	0.999	0.999	0.995	0.999	0.993
Exact recall	0.933	0.950	0.878	0.879	0.948
Overall exact *F*_1_	0.965	0.974	0.933	0.935	0.970
Partial precision	0.999	0.999	0.995	0.999	0.994
Partial recall	0.933	0.950	0.878	0.879	0.948
Overall partial *F*_1_	0.965	0.974	0.933	0.935	0.970

Table 5 shows that all models demonstrated good entity recognition and entity type classification in the test set. Almost all entity types had both exact and partial *F*_1_-scores over 0.800, except for “Unit of Measure” and, in the specific case of ThaiBERT, “Unit of presentation.” ClinicalBERT consistently outperformed the other models across most entity types, achieving the highest scores. Notably, it scored 0.933 (both exact and partial *F*_1_-scores) for “Decimal,” and 0.927 (both exact and partial *F*_1_-scores) for “Time patterns.” Its performance on “Substance” and “Route of administration” was also strong, with scores of 0.926/0.928 and 0.891/0.895, respectively. ThaiBERT followed closely with high overall *F*_1_-scores (0.969 exact, 0.970 partial), yet it displayed uneven performance; while competitive in “Route of administration” (0.890), it scored significantly lower in “Unit of presentation” (0.398) compared to the ~0.88 average of other models. BioClinical BERT showed robust performance, with exact and partial *F*_1_-scores of 0.911 and 0.914 for “Substance,” respectively, and scores of 0.880 and 0.885 for “Route of administration.” Similarly, MultilingualBERT proved effective, particularly in extracting “Decimal” (0.930) and “Time patterns” (0.926), comparable to the top performers. PubMedBERT, while showing comparable results, lagged slightly behind, particularly in handling “Substance” (0.782 exact, 0.791 partial) and “Time patterns” (0.846 exact, 0.847 partial). Its performance on “Decimal” and “Route of administration” was closer to that of BioClinical BERT, with scores in the mid to high 0.80s. Overall, ClinicalBERT’s exact and partial *F*_1_-scores of 0.973 across all entity types indicate its superior efficacy in accurately identifying and classifying clinical entities in the dataset. The full details of performance matrices and model comparisons on the fine-tuning and validation datasets are shown in Multimedia Appendix 1 (Table S1-S9).

**Table 5.** Comparison of *F*_1_-scores across BioClinicalBERT, ClinicalBERT, PubMedBERT, MultilingualBERT, and ThaiBERT on the test set.

Entity type	BioClinicalBERT		ClinicalBERT		PubMedBERT		MultilingualBERT		ThaiBERT	
	Partial *F*_1_	Exact *F*_1_	Exact *F*_1_	Partial *F*_1_	Exact *F*_1_	Partial *F*_1_	Exact *F*_1_	Partial *F*_1_	Exact *F*_1_	Partial *F*_1_
Substance	0.911	0.914	0.926	0.928	0.782	0.791	0.909	0.912	0.757	0.768
Decimal	0.926	0.926	0.933	0.933	0.874	0.874	0.930	0.930	0.883	0.884
Route of administration	0.880	0.885	0.891	0.895	0.865	0.869	0.875	0.880	0.890	0.895
Time patterns	0.918	0.918	0.927	0.927	0.846	0.847	0.926	0.926	0.824	0.828
Unit of presentation	0.888	0.888	0.892	0.892	0.888	0.888	0.873	0.876	0.398	0.411
Unit of measure	0.692	0.692	0.692	0.692	0.607	0.607	0.667	0.667	0.623	0.623
Overall	0.968	0.968	0.973	0.973	0.925	0.925	0.931	0.932	0.969	0.970

Table 6 shows the statistical validation of the performance differences. We conducted pairwise comparisons of exact performance using a 2-proportion test with Bonferroni correction. The analysis confirms that ClinicalBERT was statistically superior to all other models in overall performance (*P*<.05). ClinicalBERT significantly outperformed both PubMedBERT and ThaiBERT across nearly all specific entity types. BioClinicalBERT also achieved significantly higher overall performance compared to PubMedBERT, MultilingualBERT, and ThaiBERT. BioClinicalBERT surpassed ThaiBERT in every entity type. While ThaiBERT achieved statistically higher overall performance compared to both PubMedBERT and MultilingualBERT, the pairwise breakdown reveals significant deficiencies in specific categories. For instance, ThaiBERT performed significantly worse than MultilingualBERT in “Substance,” “Decimal,” and “Time patterns,” and significantly worse than PubMedBERT in “Unit of presentation.” The comprehensive details regarding performance metrics and model comparisons on the test dataset are available in Multimedia Appendix 1 (Tables S10-S25).

**Table 6.** League table of partial *F*_1_-score comparison by entity types among BioClinicalBERT, ClinicalBERT, PubMedBERT, MultilingualBERT, and ThaiBERT on the test dataset.^a^

Models	BioClinicalBERT	ClinicalBERT	PubMedBERT	MultilingualBERT	ThaiBERT
BioClinicalBERT	—^b^	↓ Overall	↑ Overall ↑ Substance ↑ Decimal ↑ Time patterns	↑ Overall	↑ Overall ↑ Substance ↑ Decimal ↑ Time patterns ↑ Unit of presentation
ClinicalBERT	↑ Overall	—	↑ Overall ↑ Substance ↑ Decimal ↑ Time patterns	↑ Overall	↑ Overall ↑ Substance ↑ Decimal ↑ Time patterns ↑ Unit of presentation
PubMedBERT	↓ Overall ↓ Substance ↓ Time patterns	↓ Overall ↓ Substance ↓ Time patterns	—	↓ Overall ↓ Substance ↓ Decimal ↓ Time patterns	↓ Overall ↑ Unit of presentation
MultilingualBERT	↓ Overall	↓ Overall	↑ Overall ↑ Substance ↑ Time patterns)	—	↓ Overall ↑ Substance ↑ Decimal ↑ Time patterns
ThaiBERT	↓ Substance ↓ Decimal ↓ Time patterns ↓ Unit of presentation	↓ Overall ↓ Substance ↓ Decimal ↓ Time patterns ↓ Unit of presentation	↓ Unit of presentation ↑ Overall	↓ Substance ↓ Decimal ↓ Time patterns ↓ Unit of presentation ↑ Overall	—

a The 10 pairwise (5 models) comparisons were performed using a 2-proportion test with a corrected *P*-value (2-sided) calculated using the Bonferroni method. A corrected *P* value <.05 is considered statistically significant. ↑ indicates the row model performs statistically better than the column model, and ↓ indicates the row model performs statistically worse than the column model.

b Not applicable.

## Discussion

### Principal Findings

Health care has undergone significant advancements due to EHRs, shifting from paper-based records to digital systems, which improve data access and management [34]. Ideally, EHRs reduce administrative burdens, allowing health care professionals to focus more on patient care and enabling advanced data analytics to identify trends, calculate risks, and improve treatment protocols [35]. Nevertheless, many of the currently used EHR systems are not much more than substitutes for paper charts, and most information is locked within unstructured narratives. This is an unsatisfactory situation, all the more so because much effort has been invested in the development of semantic standards such as SNOMED-CT and FHIR. Increasingly based on a formal framework, these standards bear the potential to standardize clinical information, thus supporting data interoperability with the goal of improving continuity of care and ensuring data accuracy and consistency, which is essential for developing reliable clinical applications [36].

There have been many attempts to enhance health care data by bridging the gap between clinical narratives and structured, standardized data representation by leveraging clinical NLP [37]. A slow but steady evolution of the state-of-the-art in clinical IE technologies has been witnessed through competitions and shared tasks such as the i2b2 (Integrating Biology and the Bedside) challenges held in 2010, 2011, 2012, and 2018 [12,38,39]. These competitions have promoted the public sharing of valuable datasets, such as MIMIC-III and CanTeMiST [40,41]. Many techniques have evolved over the past 2 decades, utilizing predefined lists of terms to identify meaningful words and word sequences in clinical narratives and to assign terminology codes to them [11]. Rule-based approaches, relying on rules crafted by domain experts, as well as machine learning, have demonstrated their potential [42,43]. Particularly, CRFs have gained popularity for sequence labeling tasks in clinical NER [44]. More recently, deep neural networks have revolutionized machine learning and its use in (clinical) NLP scenarios, particularly with transformer-based models such as BERT. Models such as bioClinicalBERT, ClinicalBERT, and PubMedBERT, pretrained on BERT’s architecture using large biomedical corpora, have shown promising performance in representing clinical domain text data, NER, and RE tasks [25,27,28,45].

This is why we proposed an approach that uses these models on the one hand but, on the other hand, emphasizes the target structures, which required a careful analysis of the domain and existing ontological representations, such as SNOMED-CT and web ontology language, in concert with information templates such as HL7-FHIR, which provide context for the patient-level instances of ontology concepts. Accordingly, guidelines were established for representing medication information, which were used to guide our annotation process. From the annotated medication corpus, exhibiting an idiosyncratic mixture of English and Thai language elements, fine-tuning, validation, and test data were obtained to compare the performance of different pretrained DL models for medication statement annotations. All models exhibited impressive performance metrics, with ClinicalBERT consistently outperforming the other 2 models in all scenarios, indicating its robustness. Examining each of the 8 target entity types, we found “Substance,” ie, the ingredients of the drugs whose administration was described.

### Error Analysis

From the results, all models performed very well on the test dataset, with *F*_1_-scores over 0.90. ClinicalBERT demonstrates the highest discrimination performance compared to the other models, achieving *F*_1_-scores over 0.80 in all entity types except “Unit of Measure.” The performance metrics for this category were the lowest across all models. We found that the errors of both ClinicalBERT and BioClinicalBERT were primarily type confusion caused by compound tokens, eg, “100u/10ml.” The models classified these as “Decimals,” whereas the ground truth labeled them as “Units.” This misclassification can be attributed to the sub-word tokenization mechanism of BERT-based models. For a compound string like “100u/10ml,” the tokenizer splits the text into a sequence of numeric heads and unit suffixes: [“100,” “##u,” “/,” “10,” “##ml”]. Because the model encounters the explicit numeric tokens (100, 10) as the initiating elements of the sequence, it prioritizes the numeric semantics—strongly associated with the “Decimal” class during training—over the unit suffixes. Consequently, the model labels the entire span as a numerical value rather than a measurement unit, whereas human annotators prioritized the “Unit of Measure” aspect. This suggests that the lower “Unit” performance score reflects a disagreement on label granularity for compound tokens rather than a failure to extract clinical information. In contrast, we found one instance where PubMedBERT misclassified “gm”(gram)—labeled as “Unit of Measure”—as a “Substance” entity type. We believe this error is due to the typical omission of units by physicians, such as in expressions like “Amlo (5) 1*1 po pc,” where the unit is implicit, or the use of abbreviations like “ml” for milliliters and “mg” for milligrams. Improving the recognition of abbreviated and implicit units appears essential for better NER performance.

### Models Comparison

In comparing our results to other methods, we observed the following. Tao et al [44] reported a mean *F*_1_-score of 0.864 using CFR on the 2009 i2b2 dataset, while we achieved an *F*_1_-score of 0.895 with ClinicalBERT. Tao et al [44] reached a higher “Route of administration” *F*_1_-score of 0.909. Tu et al [46] used bidirectional long short-term memory-CRF on the 2009 i2b2 dataset and achieved a weighted average *F*_1_-score of 0.985 overall, exceeding 0.70 for the medication entity type. This demonstrates similar performance to our models. Li et al [47] evaluated pretrained language models for NER in clinical trial eligibility criteria using multiple corpora (ElilE, Covance, and Chia) in the clinical domain, focusing on a broad range of entity types such as Condition, Drug, Qualifier, Measurement, Procedure, Observation, Temporal Measurement, Anatomic Location, Negation, and several others. They found that the PubMedBERT model performed best with *F*_1_-scores of 0.715, 0.836, and 0.622 for the 3 corpora, respectively [47]. A similar study by Narayanan et al [48] also used BioClinicalBERT and PubMedBERT to extract medication information and adverse events from n2n2 2018, which included 9 different entity types for medication data. Their MT-NER-PMB model achieved strict and relaxed *F*_1_-scores of 0.809/0.910 for n2c2 2018 and 0.809/0.910 for n2c2 2009. Looking at the performance of each class, MT-NER-PMB had a medication/drug entity type partial *F*_1_-score of 0.935, whereas our fine-tuned ClinicalBERT showed a partial *F*_1_-score of 0.928. However, our study uses different labels for some entity types due to our concern of referring to international standards SNOMED-CT and FHIR instead of proposing our own annotation scheme. For example, our “Decimal” entity type, which achieved a partial *F*_1_-score of 0.933, could refer to dosage and dose quantity, with our “Time patterns” entity type referring to frequency and duration. These results are comparable to MT-NER-PMB’s performance of “Dosage” (0.9438), “Duration” (0.8719), and “Form” (0.9612). Gou et al [49] demonstrated their best NER model using RoBERTa, achieving a 0.926 microaveraged *F*_1_-score for medication event classification on the n2c2 2018 dataset. In comparison, our model showed an overall partial *F*_1_-score of 0.973, indicating superior performance in NER regarding the identification and classification of medication-related text spans in clinical narratives. Furthermore, our study extends these comparisons by evaluating the impact of native language pretraining on code-switched clinical text. While it might be hypothesized that ThaiBERT or MultilingualBERT would outperform English-only models due to their correct tokenization of Thai script, our results indicate otherwise. ClinicalBERT consistently outperformed both ThaiBERT and MultilingualBERT. This finding can be attributed to the linguistic structure of Thai discharge summaries: while the narrative flow involves Thai grammar and instructions (eg, “กินวัน จันทร์ พุธ ศุกร์”), the core medical entities—such as medication names, routes, and dosage abbreviations—are predominantly written in English or Latin (eg, “Simvastatin,” “po,” “bid”). Consequently, even though English-specific models treat Thai characters as out-of-vocabulary tokens, they effectively utilize the robust English medical terminology as semantic anchors. This suggests that for this specific task, biomedical domain pretraining is more critical than native vocabulary coverage.

Since 2022, the success of generative AI based on large language models, with its flagship ChatGPT, has demonstrated great performance on various tasks in NLP. It is, therefore, interesting to mention the approach regarding their NER performance, particularly in terms of the shift from classical annotation to prompt engineering, without the need for large training datasets. In a recent study, we investigated the use of multiple prompt commands in ChatGPT to perform NER tasks on Thai medication prescription data from the same hospital as our study [16]. This study yielded an average strict precision and recall of 0.84 and 0.75, respectively, for the best-performing NER prompt. Although this ChatGPT zero-shot approach showed promising NER performance, the model’s misrecognition, evidenced by poor recall (0.66) in the entity type of instructions, raised a major concern about this approach for medication data extraction. This entity type misclassification could result in missing important parts of medication prescriptions, particularly dose quantity, timing, and route. Hu et al [50] also explored using ChatGPT to extract medical problems, treatments, and tests from synthetic discharge summaries based on the 2010 i2b2 challenge guidelines. Using different prompts and versions of ChatGPT, they reported *F*_1_-scores of 0.436 (strict) and 0.719 (partial) for ChatGPT 3.5, and 0.526 (strict) and 0.838 (partial) for ChatGPT 4 [51].

While Generative AIs have proven useful for NER tasks, we believe that pretrained DL models still hold some advantage over Generative AIs, particularly in terms of patient data privacy and safety protocols. Utilizing commercial Generative AI application programming interfaces requires transmitting sensitive discharge summaries to external servers, which often violates strict data governance and patient confidentiality standards. In contrast, DL models are compact enough to be deployed entirely on-premises, ensuring that protected health information never leaves the hospital’s secure infrastructure. Furthermore, as discriminative classifiers, BERT models are constrained to extracting existing text, inherently minimizing the risk of “hallucination”—the generation of plausible but nonexistent medical facts—which remains a critical safety liability for Generative AIs [52-54]. Therefore, while Generative AIs offer broad reasoning capabilities, fine-tuned transformers remain the most robust, privacy-compliant solution for high-stakes clinical entity extraction. Future work may explore hybrid systems that leverage the reasoning capabilities of Generative AIs—either through secure APIs or via local, on-premises model deployments—while maintaining the strict data safety constraints of the hospital environment.

### Conclusion

Our study demonstrates that fine-tuned pretrained transformer models—especially those with biomedical domain pretraining—have high clinical NER performance for extracting structured medication statements from clinical narratives in a Thai hospital.

While the models achieved high *F*_1_-scores across various entity types, certain categories, such as the “Unit of Measure,” demonstrated the need for future improvement. Compared to other methods, our models exhibit competitive or superior performance, particularly in medication entity classification. The core strength of our study lies in the annotation and use of our data to fine-tune, validate, and test the model, specifically tailored for a Thai hospital setting that features Thai-English code-switched narrative clinical text. Our results also validate that English-pretrained biomedical models are a viable and robust solution for this setting, effectively utilizing English medical terminology as semantic anchors even within Thai grammatical structures.

Unlike previous studies using public datasets like n2c2 2009 or i2b2 2009, we leverage an ontology-based approach, which adds value by enhancing precision, reducing ambiguity, and enabling better integration and interoperability with standard terminology systems. This approach also provides a structured framework for handling context-specific terminology. However, there is a gap in utilizing our annotated dataset for RE tasks. Expanding the dataset to include more diverse entities, such as diseases, procedures, and temporal data, is necessary to enhance the model’s capacity to extract complex medical information.

Nevertheless, we acknowledge that the current study represents only the first stage of semantic interoperability. While our models achieve high performance in the NER task, the next critical step is normalizing these entities to their specific fine-grained SNOMED-CT codes and linking their relations together. Future work will need to leverage the granular annotations preserved in our dataset to develop downstream MCN and RE models to enhance the model’s capacity to extract complex medical information.

## Supplementary material

10.2196/82685 Multimedia Appendix 1 Exhaustive statistical analyses and granular performance metrics of the five evaluated transformer models.

## Abbreviations

AI: artificial intelligence
BERT: Bidirectional Encoder Representations From Transformers
BFO: basic foundational ontology
CRF: conditional random field
DL: deep learning
EHR: electronic health record
HL7 FHIR: Health Level Seven Fast Healthcare Interoperability Resources
IE: information extraction
MCN: medical concept normalization
NER: named entity recognition
NLP: natural language processing
RE: relation extraction
SNOMED-CT: Systematized Nomenclature of Medicine-Clinical Terms
