# Supplementary material for: Ontology-Based Medication Named Entity Recognition Using Pretrained Transformer Models From a Thai Hospital: Model Fine-Tuning and Validation Study
Source: JMIR Form Res. 2026 Mar 20;10:e82685. doi: 10.2196/82685 (PMC13004563; doi:10.2196/82685)
Supplement: Multimedia Appendix 1 [file formative-v10-e82685-s001.docx]

**Table S1.** League table of Exact performance of Overall entities comparison among for BioClinicalBERT, ClinicalBERT, PubMedBERT MultilingualBERT, and ThaiBERT on the fine-tuning and validation datasets.

| **Models** | **BioClinical**  **BERT** | **Clinical**  **BERT** | **PubMed**  **BERT** | **Multilingual**  **BERT** | **Thai**  **BERT** |
| --- | --- | --- | --- | --- | --- |
| **BioClinical**  **BERT** | — | ↓  (Recall, F1) | ↑  (All) | ↑  (Recall, F1) | ↑  (Precision) |
| **Clinical**  **BERT** | ↑  (Recall, F1) | — | ↑  (All) | ↑  (All) | ↑  (Precision, F1) |
| **PubMed**  **BERT** | ↓  (All) | ↓  (All) | — | ↓  (Precision) | ↓  (All) |
| **Multilingual**  **BERT** | ↓  (Recall, F1) | ↓  (All) | ↑  (Precision) | — | ↓  (All) |
| **Thai**  **BERT** | ↓  (Precision) | ↓  (Precision, F1) | ↓  (Precision) | ↓  (Precision) | — |
|  | ↑  (Recall) |  | ↑  (Recall, F1) | ↑  (Recall, F1) |  |

The ten pairwise (five models) comparisons were performed using two-proportion test with corrected p-value (two-sided) using Bonferroni method. A corrected p-value <0.05 is considered statistically significance.Green with ↑ indicates the row model is statistically better than the column model. Red with ↓ the indicates row model is statistically worse than the column model.

**Table S2.** League table of Partial performance of Overall entities comparison among for BioClinicalBERT, ClinicalBERT, PubMedBERT MultilingualBERT, and ThaiBERT on the fine-tuning and validation datasets.

| **Models** | **BioClinical**  **BERT** | **Clinical**  **BERT** | **PubMed**  **BERT** | **Multilingual**  **BERT** | **Thai**  **BERT** |
| --- | --- | --- | --- | --- | --- |
| **BioClinical**  **BERT** | — | ↓  (Recall, F1) | ↑  (All) | ↑  (Recall, F1) | ↑  (Precision) |
| **Clinical**  **BERT** | ↑  (Recall, F1) | — | ↑  (All) | ↑  (All) | ↑  (Precision, F1) |
| **PubMed**  **BERT** | ↓  (All) | ↓  (All) | — | ↓  (Precision) | ↓  (All) |
| **Multilingual**  **BERT** | ↓  (Recall, F1) | ↓  (All) | ↑  (Precision) | — | ↓  (All) |
| **Thai**  **BERT** | ↓  (Precision) | ↓  (Precision, F1) | ↓  (Precision) | ↓  (Precision) | — |
|  | ↑  (Recall) |  | ↑  (Recall, F1) | ↑  (Recall, F1) |  |

The ten pairwise (five models) comparisons were performed using two-proportion test with corrected p-value (two-sided) using Bonferroni method. A corrected p-value <0.05 is considered statistically significance.Green with ↑ indicates the row model is statistically better than the column model. Red with ↓ the indicates row model is statistically worse than the column model.

**Table S3.** Corrected p-values from Exact Recall of Overall entities comparison among for BioClinicalBERT, ClinicalBERT, PubMedBERT MultilingualBERT, and ThaiBERT on the fine-tuning and validation datasets.

| **Models**  **(Exact Recall)** | **BioClinical**  **BERT**  **(0.933)** | **Clinical**  **BERT**  **(0.950)** | **PubMed**  **BERT**  **(0.878)** | **Multilingual**  **BERT**  **(0.879)** | **Thai**  **BERT**  **(0.948)** |
| --- | --- | --- | --- | --- | --- |
| **BioClinical**  **BERT**  **(0.933)** | — | ↓  < 0.001 | ↑  < 0.001 | ↑  < 0.001 | ↓  < 0.001 |
| **Clinical**  **BERT**  **(0.950)** | ↑  < 0.001 | — | ↑  < 0.001 | ↑  < 0.001 | 1.000 |
| **PubMed**  **BERT**  **(0.878)** | ↓  < 0.001 | ↓  < 0.001 | — | 1.000 | ↓  < 0.001 |
| **Multilingual**  **BERT**  **(0.879)** | ↓  < 0.001 | ↓  < 0.001 | 1.000 | — | ↓  < 0.001 |
| **Thai**  **BERT**  **(0.948)** | ↑  < 0.001 | 1.000 | ↑  < 0.001 | ↑  < 0.001 | — |

**Table S4.** Corrected p-values from Partial Recall of Overall entities comparison among for BioClinicalBERT, ClinicalBERT, PubMedBERT MultilingualBERT, and ThaiBERT on the fine-tuning and validation datasets.

| **Models**  **(Partial Recall)** | **BioClinical**  **BERT**  **(0.933)** | **Clinical**  **BERT**  **(0.950)** | **PubMed**  **BERT**  **(0.878)** | **Multilingual**  **BERT**  **(0.879)** | **Thai**  **BERT**  **(0.948)** |
| --- | --- | --- | --- | --- | --- |
| **BioClinical**  **BERT**  **(0.933)** | — | ↓  < 0.001 | ↑  < 0.001 | ↑  < 0.001 | ↓  < 0.001 |
| **Clinical**  **BERT**  **(0.950)** | ↑  < 0.001 | — | ↑  < 0.001 | ↑  < 0.001 | 1.000 |
| **PubMed**  **BERT**  **(0.878)** | ↓  < 0.001 | ↓  < 0.001 | — | 1.000 | ↓  < 0.001 |
| **Multilingual**  **BERT**  **(0.879)** | ↓  < 0.001 | ↓  < 0.001 | 1.000 | — | ↓  < 0.001 |
| **Thai**  **BERT**  **(0.948)** | ↑  < 0.001 | 1.000 | ↑  < 0.001 | ↑  < 0.001 | — |

The ten pairwise (five models) comparisons were performed using two-proportion test with corrected p-value (two-sided) using Bonferroni method. Green with ↑ indicates the row model is statistically better than the column model. Red with ↓ the indicates row model is statistically worse than the column model. Gray indicates no statistically significant difference (p-value ≥0.05 after correction).

**Table S5.** Corrected p-values from Exact Precision of Overall entities comparison among for BioClinicalBERT, ClinicalBERT, PubMedBERT MultilingualBERT, and ThaiBERT on the fine-tuning and validation datasets.

| **Models**  **(Exact precision)** | **BioClinical**  **BERT**  **(0.999)** | **Clinical**  **BERT**  **(0.999)** | **PubMed**  **BERT**  **(0.996)** | **Multilingual**  **BERT**  **(0.999)** | **Thai**  **BERT**  **(0.993)** |
| --- | --- | --- | --- | --- | --- |
| **BioClinical**  **BERT**  **(0.999)** | — | 1.000 | ↑  < 0.001 | 1.000 | ↑  < 0.001 |
| **Clinical**  **BERT**  **(0.999)** | 1.000 | — | ↑  < 0.001 | 1.000 | ↑  < 0.001 |
| **PubMed**  **BERT**  **(0.996)** | ↓  < 0.001 | ↓  < 0.001 | — | ↓  < 0.001 | ↑  < 0.001 |
| **Multilingual**  **BERT**  **(0.999)** | 1.000 | 1.000 | ↑  < 0.001 | — | ↑  < 0.001 |
| **Thai**  **BERT**  **(0.993)** | ↓  < 0.001 | ↓  < 0.001 | ↓  < 0.001 | ↓  < 0.001 | — |

**Table S6.** Corrected p-values from Partial Precision of Overall entities comparison among for BioClinicalBERT, ClinicalBERT, PubMedBERT MultilingualBERT, and ThaiBERT on the fine-tuning and validation datasets.

| **Models**  **(Partial precision)** | **BioClinical**  **BERT**  **(0.999)** | **Clinical**  **BERT**  **(0.999)** | **PubMed**  **BERT**  **(0.996)** | **Multilingual**  **BERT**  **(0.999)** | **Thai**  **BERT**  **(0.994)** |
| --- | --- | --- | --- | --- | --- |
| **BioClinical**  **BERT**  **(0.999)** | — | 1.000 | ↑  < 0.001 | 1.000 | ↑  < 0.001 |
| **Clinical**  **BERT**  **(0.999)** | 1.000 | — | ↑  < 0.001 | 1.000 | ↑  < 0.001 |
| **PubMed**  **BERT**  **(0.996)** | ↓  < 0.001 | ↓  < 0.001 | — | ↓  < 0.001 | ↑  < 0.001 |
| **Multilingual**  **BERT**  **(0.999)** | 1.000 | 1.000 | ↑  < 0.001 | — | ↑  < 0.001 |
| **Thai**  **BERT**  **(0.994)** | ↓  < 0.001 | ↓  < 0.001 | ↓  < 0.001 | ↓  < 0.001 | — |

The ten pairwise (five models) comparisons were performed using two-proportion test with corrected p-value (two-sided) using Bonferroni method. Green with ↑ indicates the row model is statistically better than the column model. Red with ↓ the indicates row model is statistically worse than the column model. Gray indicates no statistically significant difference (p-value ≥0.05 after correction).

**Table S7.** Corrected p-values from Exact F1-score of Overall entities comparison among for BioClinicalBERT, ClinicalBERT, PubMedBERT MultilingualBERT, and ThaiBERT on the fine-tuning and validation datasets.

| **Models**  **(Exact F1-score)** | **BioClinical**  **BERT**  **(0.965)** | **Clinical**  **BERT**  **(0.974)** | **PubMed**  **BERT**  **(0.933)** | **Multilingual**  **BERT**  **(0.935)** | **Thai**  **BERT**  **(0.970)** |
| --- | --- | --- | --- | --- | --- |
| **BioClinical**  **BERT**  **(0.965)** | — | ↓  < 0.001 | ↑  < 0.001 | ↑  < 0.001 | ↓  0.002 |
| **Clinical**  **BERT**  **(0.974)** | ↑  < 0.001 | — | ↑  < 0.001 | ↑  < 0.001 | ↑  0.010 |
| **PubMed**  **BERT**  **(0.933)** | ↓  < 0.001 | ↓  < 0.001 | — | 1.000 | ↓  < 0.001 |
| **Multilingual**  **BERT**  **(0.935)** | ↓  < 0.001 | ↓  < 0.001 | 1.000 | — | ↓  < 0.001 |
| **Thai**  **BERT**  **(0.970)** | ↑  0.002 | ↓  0.010 | ↑  < 0.001 | ↑  < 0.001 | — |

**Table S8.** Corrected p-values from Partial F1-score of Overall entities comparison among for BioClinicalBERT, ClinicalBERT, PubMedBERT MultilingualBERT, and ThaiBERT on the fine-tuning and validation datasets.

| **Models**  **(Partial F1-score)** | **BioClinical**  **BERT**  **(0.965)** | **Clinical**  **BERT**  **(0.974)** | **PubMed**  **BERT**  **(0.933)** | **Multilingual**  **BERT**  **(0.935)** | **Thai**  **BERT**  **(0.970)** |
| --- | --- | --- | --- | --- | --- |
| **BioClinical**  **BERT**  **(0.965)** | — | ↓  < 0.001 | ↑  < 0.001 | ↑  < 0.001 | ↓  0.002 |
| **Clinical**  **BERT**  **(0.974)** | ↑  < 0.001 | — | ↑  < 0.001 | ↑  < 0.001 | ↑  0.010 |
| **PubMed**  **BERT**  **(0.933)** | ↓  < 0.001 | ↓  < 0.001 | — | 1.000 | ↓  < 0.001 |
| **Multilingual**  **BERT**  **(0.935)** | ↓  < 0.001 | ↓  < 0.001 | 1.000 | — | ↓  < 0.001 |
| **Thai**  **BERT**  **(0.970)** | ↑  0.002 | ↓  0.010 | ↑  < 0.001 | ↑  < 0.001 | — |

The ten pairwise (five models) comparisons were performed using two-proportion test with corrected p-value (two-sided) using Bonferroni method. Green with ↑ indicates the row model is statistically better than the column model. Red with ↓ the indicates row model is statistically worse than the column model. Gray indicates no statistically significant difference (p-value ≥0.05 after correction).

**Table S9.** Detials performance metrics on the fine-tuning and validation datasets

| Models | Entity  types | Exact  Precision | Exact  Recall | Exact  F1 | Partial  Precision | Partial  Recall | Partial  F1 |
| --- | --- | --- | --- | --- | --- | --- | --- |
| **BioClinical**  **BERT** | O | 1.000 | 0.946 | 0.972 | 1.000 | 0.946 | 0.972 |
|  | SUB | 0.957 | 0.656 | 0.779 | 0.962 | 0.660 | 0.783 |
|  | DEC | 0.990 | 0.676 | 0.803 | 0.990 | 0.676 | 0.803 |
|  | ROU | 0.982 | 0.664 | 0.792 | 0.982 | 0.664 | 0.792 |
|  | TIM | 0.983 | 0.629 | 0.768 | 0.984 | 0.630 | 0.768 |
|  | UNI | 1.000 | 0.850 | 0.919 | 1.000 | 0.850 | 0.919 |
|  | MEA | 1.000 | 0.450 | 0.621 | 1.000 | 0.450 | 0.621 |
|  | Overall | 0.999 | 0.933 | 0.965 | 0.999 | 0.933 | 0.965 |
| **Clinical**  **BERT** | O | 1.000 | 0.961 | 0.980 | 1.000 | 0.961 | 0.980 |
|  | SUB | 0.961 | 0.697 | 0.808 | 0.966 | 0.701 | 0.812 |
|  | DEC | 0.992 | 0.727 | 0.839 | 0.992 | 0.727 | 0.839 |
|  | ROU | 0.992 | 0.706 | 0.825 | 0.992 | 0.706 | 0.825 |
|  | TIM | 0.965 | 0.663 | 0.786 | 0.965 | 0.663 | 0.786 |
|  | UNI | 1.000 | 0.925 | 0.961 | 1.000 | 0.925 | 0.961 |
|  | MEA | 0.955 | 0.525 | 0.677 | 0.955 | 0.525 | 0.677 |
|  | Overall | 0.999 | 0.950 | 0.974 | 0.999 | 0.950 | 0.974 |
| **PubMed**  **BERT** | O | 0.998 | 0.891 | 0.942 | 0.998 | 0.891 | 0.942 |
|  | SUB | 0.878 | 0.594 | 0.709 | 0.882 | 0.597 | 0.712 |
|  | DEC | 0.929 | 0.621 | 0.744 | 0.929 | 0.621 | 0.744 |
|  | ROU | 0.949 | 0.630 | 0.757 | 0.949 | 0.630 | 0.757 |
|  | TIM | 0.874 | 0.553 | 0.677 | 0.874 | 0.553 | 0.677 |
|  | UNI | 1.000 | 0.825 | 0.904 | 1.000 | 0.825 | 0.904 |
|  | MEA | 0.556 | 0.250 | 0.345 | 0.556 | 0.250 | 0.345 |
|  | Overall | 0.996 | 0.878 | 0.933 | 0.996 | 0.878 | 0.933 |
| **Multilingual**  **BERT** | O | 1.000 | 0.889 | 0.941 | 1.000 | 0.889 | 0.941 |
|  | SUB | 0.960 | 0.650 | 0.775 | 0.964 | 0.653 | 0.778 |
|  | DEC | 0.987 | 0.660 | 0.791 | 0.987 | 0.660 | 0.791 |
|  | ROU | 0.994 | 0.660 | 0.793 | 0.994 | 0.660 | 0.793 |
|  | TIM | 0.969 | 0.613 | 0.751 | 0.973 | 0.615 | 0.754 |
|  | UNI | 1.000 | 0.825 | 0.904 | 1.000 | 0.825 | 0.904 |
|  | MEA | 1.000 | 0.450 | 0.621 | 1.000 | 0.450 | 0.621 |
|  | Overall | 0.999 | 0.879 | 0.935 | 0.999 | 0.879 | 0.935 |
| **Thai**  **BERT** | O | 0.999 | 0.964 | 0.981 | 0.999 | 0.964 | 0.981 |
|  | SUB | 0.782 | 0.579 | 0.665 | 0.788 | 0.583 | 0.670 |
|  | DEC | 0.893 | 0.652 | 0.754 | 0.895 | 0.653 | 0.755 |
|  | ROU | 0.976 | 0.718 | 0.827 | 0.976 | 0.719 | 0.828 |
|  | TIM | 0.749 | 0.517 | 0.612 | 0.764 | 0.527 | 0.624 |
|  | UNI | 0.500 | 0.413 | 0.452 | 0.500 | 0.413 | 0.452 |
|  | MEA | 0.591 | 0.325 | 0.419 | 0.591 | 0.325 | 0.419 |
|  | Overall | 0.993 | 0.948 | 0.970 | 0.994 | 0.948 | 0.970 |

**Abbreviations:** O, Outside; SUB, Substance; DEC, Decimal; ROU, Route of admisnistration; TIM, Time presentation; UNI, Unit of presentation; MEA, Unit of measure.

**Table S10.** League table of Exact performance comparison by entity types among BioClinicalBERT, ClinicalBERT, PubMedBERT MultilingualBERT, and ThaiBERT on the test datasets.

| **Models** | **BioClinical**  **BERT** | **Clinical**  **BERT** | **PubMed**  **BERT** | **Multilingual**  **BERT** | **Thai**  **BERT** |
| --- | --- | --- | --- | --- | --- |
| **BioClinical**  **BERT** | — | ↓  (Overall) | ↑  (Overall,  Substance, Decimal,  Time patterns) | ↑  (Overall) | ↑  (Overall,  Substance, Decimal,  Time patterns,  Unit of presentation) |
| **Clinical**  **BERT** | ↑  (Overall) | — | ↑  (Overall,  Substance, Decimal,  Time patterns) | ↑  (Overall) | ↑  (Overall,  Substance,  Decimal,  Time patterns,  Unit of presentation) |
| **PubMed**  **BERT** | ↓  (Overall,  Substance,  Time patterns) | ↓  (Overall,  Substance,  Time patterns) | — | ↓  (Overall, Substance, Decimal,  Time patterns) | ↓  (Overall) |
|  |  |  |  |  | ↑  (Unit of presentation) |
| **Multilingual**  **BERT** | ↓  (Overall) | ↓  (Overall) | ↑  (Overall, Substance,  Time patterns) | — | ↓  (Overall) |
|  |  |  |  |  | ↑  (Substance, Decimal,  Time patterns) |
| **Thai**  **BERT** | ↓  (Substance,  Decimal,  Time patterns,  Unit of presentation) | ↓  (Overall,  Substance,  Decimal,  Time patterns,  Unit of presentation) | ↓  (Unit of presentation) | ↓  (Substance, Decimal,  Time patterns, Unit of presentation) | — |
|  |  |  | ↑  (Overall) | ↑  (Overall) |  |

The ten pairwise (five models) comparisons were performed using two-proportion test with corrected p-value (two-sided) using Bonferroni method. A corrected p-value <0.05 is considered statistically significance.Green with ↑ indicates the row model is statistically better than the column model. Red with ↓ the indicates row model is statistically worse than the column model.

**Table S10.** League table of Partial performance comparison by entity types among BioClinicalBERT, ClinicalBERT, PubMedBERT MultilingualBERT, and ThaiBERT on the test datasets.

| **Models** | **BioClinical**  **BERT** | **Clinical**  **BERT** | **PubMed**  **BERT** | **Multilingual**  **BERT** | **Thai**  **BERT** |
| --- | --- | --- | --- | --- | --- |
| **BioClinical**  **BERT** | — | ↓  (Overall) | ↑  (Overall,  Substance, Decimal,  Time patterns) | ↑  (Overall) | ↑  (Overall,  Substance, Decimal,  Time patterns,  Unit of presentation) |
| **Clinical**  **BERT** | ↑  (Overall) | — | ↑  (Overall,  Substance, Decimal,  Time patterns) | ↑  (Overall) | ↑  (Overall,  Substance,  Decimal,  Time patterns,  Unit of presentation) |
| **PubMed**  **BERT** | ↓  (Overall,  Substance,  Time patterns) | ↓  (Overall,  Substance,  Time patterns) | — | ↓  (Overall, Substance, Decimal,  Time patterns) | ↓  (Overall) |
|  |  |  |  |  | ↑  (Unit of presentation) |
| **Multilingual**  **BERT** | ↓  (Overall) | ↓  (Overall) | ↑  (Overall, Substance,  Time patterns) | — | ↓  (Overall) |
|  |  |  |  |  | ↑  (Substance, Decimal,  Time patterns) |
| **Thai**  **BERT** | ↓  (Substance,  Decimal,  Time patterns,  Unit of presentation) | ↓  (Overall,  Substance,  Decimal,  Time patterns,  Unit of presentation) | ↓  (Unit of presentation) | ↓  (Substance, Decimal,  Time patterns, Unit of presentation) | — |
|  |  |  | ↑  (Overall) | ↑  (Overall) |  |

The ten pairwise (five models) comparisons were performed using two-proportion test with corrected p-value (two-sided) using Bonferroni method. A corrected p-value <0.05 is considered statistically significance.Green with ↑ indicates the row model is statistically better than the column model. Red with ↓ the indicates row model is statistically worse than the column model.

**Table S11.** Corrected p-values from Exact F1-score of Overall entities comparison among for BioClinicalBERT, ClinicalBERT, PubMedBERT MultilingualBERT, and ThaiBERT on the test datasets.

| **Models**  **(Exact F1-score)** | **BioClinical**  **BERT**  **(0.968)** | **Clinical**  **BERT**  **(0.973)** | **PubMed**  **BERT**  **(0.925)** | **Multilingual**  **BERT**  **(0.931)** | **Thai**  **BERT**  **(0.969)** |
| --- | --- | --- | --- | --- | --- |
| **BioClinical**  **BERT**  **(0.968)** | — | ↓  < 0.001 | ↑  < 0.001 | ↑  < 0.001 | 1.000 |
| **Clinical**  **BERT**  **(0.973)** | ↑  < 0.001 | — | ↑  < 0.001 | ↑  < 0.001 | ↑  0.001 |
| **PubMed**  **BERT**  **(0.925)** | ↓  < 0.001 | ↓  < 0.001 | — | ↓  0.004 | ↓  < 0.001 |
| **Multilingual**  **BERT**  **(0.931)** | ↓  < 0.001 | ↓  < 0.001 | ↑  0.004 | — | ↓  < 0.001 |
| **Thai**  **BERT**  **(0.969)** | 1.000 | ↓  0.001 | ↑  < 0.001 | ↑  < 0.001 | — |

**Table S12.** Corrected p-values from Partial F1-score of Overall entities comparison among for BioClinicalBERT, ClinicalBERT, PubMedBERT MultilingualBERT, and ThaiBERT on the test datasets.

| **Models**  **(Partial** **F1-score)** | **BioClinical**  **BERT**  **(0.969)** | **Clinical**  **BERT**  **(0.973)** | **PubMed**  **BERT**  **(0.926)** | **Multilingual**  **BERT**  **(0.932)** | **Thai**  **BERT**  **(0.970)** |
| --- | --- | --- | --- | --- | --- |
| **BioClinical**  **BERT**  **(0.969)** | — | ↓  < 0.001 | ↑  < 0.001 | ↑  < 0.001 | 1.000 |
| **Clinical**  **BERT**  **(0.973)** | ↑  < 0.001 | — | ↑  < 0.001 | ↑  < 0.001 | ↑  0.002 |
| **PubMed**  **BERT**  **(0.926)** | ↓  < 0.001 | ↓  < 0.001 | — | ↓  0.005 | ↓  < 0.001 |
| **Multilingual**  **BERT**  **(0.932)** | ↓  < 0.001 | ↓  < 0.001 | ↑  0.005 | — | ↓  < 0.001 |
| **Thai**  **BERT**  **(0.970)** | 1.000 | ↓  0.002 | ↑  < 0.001 | ↑  < 0.001 | — |

The ten pairwise (five models) comparisons were performed using two-proportion test with corrected p-value (two-sided) using Bonferroni method. Green with ↑ indicates the row model is statistically better than the column model. Red with ↓ the indicates row model is statistically worse than the column model. Gray indicates no statistically significant difference (p-value ≥0.05 after correction).

**Table S13.** Corrected p-values from Exact F1-score of Substance entity comparison among for BioClinicalBERT, ClinicalBERT, PubMedBERT MultilingualBERT, and ThaiBERT on the test datasets.

| **Models**  **(Exact** **F1-score)** | **BioClinical**  **BERT**  **(0.911)** | **Clinical**  **BERT**  **(0.927)** | **PubMed**  **BERT**  **(0.783)** | **Multilingual**  **BERT**  **(0.909)** | **Thai**  **BERT**  **(0.757)** |
| --- | --- | --- | --- | --- | --- |
| **BioClinical**  **BERT**  **(0.911)** | — | 1.000 | ↑  < 0.001 | 1.000 | ↑  < 0.001 |
| **Clinical**  **BERT**  **(0.927)** | 1.000 | — | ↑  < 0.001 | 1.000 | ↑  < 0.001 |
| **PubMed**  **BERT**  **(0.783)** | ↓  < 0.001 | ↓  < 0.001 | — | ↓  < 0.001 | 1.000 |
| **Multilingual**  **BERT**  **(0.909)** | 1.000 | 1.000 | ↑  < 0.001 | — | ↑  < 0.001 |
| **Thai**  **BERT**  **(0.757)** | ↓  < 0.001 | ↓  < 0.001 | 1.000 | ↓  < 0.001 | — |

**Table S14.** Corrected p-values from Partial F1-score of Substance entity comparison among for BioClinicalBERT, ClinicalBERT, PubMedBERT MultilingualBERT, and ThaiBERT on the test datasets.

| **Models**  **(Partial** **F1-score)** | **BioClinical**  **BERT**  **(0.914)** | **Clinical**  **BERT**  **(0.928)** | **PubMed**  **BERT**  **(0.791)** | **Multilingual**  **BERT**  **(0.912)** | **Thai**  **BERT**  **(0.768)** |
| --- | --- | --- | --- | --- | --- |
| **BioClinical**  **BERT**  **(0.914)** | — | 1.000 | ↑  < 0.001 | 1.000 | ↑  < 0.001 |
| **Clinical**  **BERT**  **(0.928)** | 1.000 | — | ↑  < 0.001 | 1.000 | ↑  < 0.001 |
| **PubMed**  **BERT**  **(0.791)** | ↓  < 0.001 | ↓  < 0.001 | — | ↓  < 0.001 | 1.000 |
| **Multilingual**  **BERT**  **(0.912)** | 1.000 | 1.000 | ↑  < 0.001 | — | ↑  < 0.001 |
| **Thai**  **BERT**  **(0.768)** | ↓  < 0.001 | ↓  < 0.001 | 1.000 | ↓  < 0.001 | — |

The ten pairwise (five models) comparisons were performed using two-proportion test with corrected p-value (two-sided) using Bonferroni method. Green with ↑ indicates the row model is statistically better than the column model. Red with ↓ the indicates row model is statistically worse than the column model. Gray indicates no statistically significant difference (p-value ≥0.05 after correction).

**Table S15.** Corrected p-values from Exact F1-score of Decimal entity comparison among for BioClinicalBERT, ClinicalBERT, PubMedBERT MultilingualBERT, and ThaiBERT on the test datasets.

| **Models**  **(Exact** **F1-score)** | **BioClinical**  **BERT**  **(0.927)** | **Clinical**  **BERT**  **(0.934)** | **PubMed**  **BERT**  **(0.874)** | **Multilingual**  **BERT**  **(0.930)** | **Thai**  **BERT**  **(0.883)** |
| --- | --- | --- | --- | --- | --- |
| **BioClinical**  **BERT**  **(0.927)** | — | 1.000 | ↑  < 0.001 | 1.000 | ↑  0.002 |
| **Clinical**  **BERT**  **(0.934)** | 1.000 | — | ↑  < 0.001 | 1.000 | ↑  < 0.001 |
| **PubMed**  **BERT**  **(0.874)** | ↓  < 0.001 | ↓  < 0.001 | — | ↓  < 0.001 | 1.000 |
| **Multilingual**  **BERT**  **(0.930)** | 1.000 | 1.000 | ↑  < 0.001 | — | ↑  < 0.001 |
| **Thai**  **BERT**  **(0.883)** | ↓  0.002 | ↓  < 0.001 | 1.000 | ↓  0.001 | — |

**Table S16.** Corrected p-values from Partial F1-score of Decimal entity comparison among for BioClinicalBERT, ClinicalBERT, PubMedBERT MultilingualBERT, and ThaiBERT on the test datasets.

| **Models**  **(Partial** **F1-score)** | **BioClinical**  **BERT**  **(0.927)** | **Clinical**  **BERT**  **(0.934)** | **PubMed**  **BERT**  **(0.874)** | **Multilingual**  **BERT**  **(0.930)** | **Thai**  **BERT**  **(0.884)** |
| --- | --- | --- | --- | --- | --- |
| **BioClinical**  **BERT**  **(0.927)** | — | 1.000 | ↑  < 0.001 | 1.000 | ↑  0.002 |
| **Clinical**  **BERT**  **(0.934)** | 1.000 | — | ↑  < 0.001 | 1.000 | ↑  < 0.001 |
| **PubMed**  **BERT**  **(0.874)** | ↓  < 0.001 | ↓  < 0.001 | — | ↓  < 0.001 | 1.000 |
| **Multilingual**  **BERT**  **(0.930)** | 1.000 | 1.000 | ↑  < 0.001 | — | ↑  < 0.001 |
| **Thai**  **BERT**  **(0.884)** | ↓  0.002 | ↓  < 0.001 | 1.000 | ↓  0.001 | — |

The ten pairwise (five models) comparisons were performed using two-proportion test with corrected p-value (two-sided) using Bonferroni method. Green with ↑ indicates the row model is statistically better than the column model. Red with ↓ the indicates row model is statistically worse than the column model. Gray indicates no statistically significant difference (p-value ≥0.05 after correction).

**Table S17.** Corrected p-values from Exact F1-score of Route of administration entity comparison among for BioClinicalBERT, ClinicalBERT, PubMedBERT MultilingualBERT, and ThaiBERT on the test datasets.

| **Models**  **(Exact** **F1-score)** | **BioClinical**  **BERT**  **(0.880)** | **Clinical**  **BERT**  **(0.891)** | **PubMed**  **BERT**  **(0. 865)** | **Multilingual**  **BERT**  **(0.875)** | **Thai**  **BERT**  **(0.890)** |
| --- | --- | --- | --- | --- | --- |
| **BioClinical**  **BERT**  **(0.880)** | — | 1.000 | 1.000 | 1.000 | 1.000 |
| **Clinical**  **BERT**  **(0.891)** | 1.000 | — | 1.000 | 1.000 | 1.000 |
| **PubMed**  **BERT**  **(0.865)** | 1.000 | 1.000 | — | 1.000 | 1.000 |
| **Multilingual**  **BERT**  **(0.875)** | 1.000 | 1.000 | 1.000 | — | 1.000 |
| **Thai**  **BERT**  **(0.890)** | 1.000 | 1.000 | 1.000 | 1.000 | — |

**Table S18.** Corrected p-values from Partial F1-score of Route of administration entity comparison among for BioClinicalBERT, ClinicalBERT, PubMedBERT MultilingualBERT, and ThaiBERT on the test datasets.

| **Models**  **(Partial** **F1-score)** | **BioClinical**  **BERT**  **(0.885)** | **Clinical**  **BERT**  **(0.896)** | **PubMed**  **BERT**  **(0.870)** | **Multilingual**  **BERT**  **(0.880)** | **Thai**  **BERT**  **(0.895)** |
| --- | --- | --- | --- | --- | --- |
| **BioClinical**  **BERT**  **(0.885)** | — | 1.000 | 1.000 | 1.000 | 1.000 |
| **Clinical**  **BERT**  **(0.896)** | 1.000 | — | 1.000 | 1.000 | 1.000 |
| **PubMed**  **BERT**  **(0.870)** | 1.000 | 1.000 | — | 1.000 | 1.000 |
| **Multilingual**  **BERT**  **(0.880)** | 1.000 | 1.000 | 1.000 | — | 1.000 |
| **Thai**  **BERT**  **(0.895)** | 1.000 | 1.000 | 1.000 | 1.000 | — |

The ten pairwise (five models) comparisons were performed using two-proportion test with corrected p-value (two-sided) using Bonferroni method. Green with ↑ indicates the row model is statistically better than the column model. Red with ↓ the indicates row model is statistically worse than the column model. Gray indicates no statistically significant difference (p-value ≥0.05 after correction).

**Table S19.** Corrected p-values from Exact F1-score of Time patterns entity comparison among for BioClinicalBERT, ClinicalBERT, PubMedBERT MultilingualBERT, and ThaiBERT on the test datasets.

| **Models**  **(Exact** **F1-score)** | **BioClinical**  **BERT**  **(0.918)** | **Clinical**  **BERT**  **(0.927)** | **PubMed**  **BERT**  **(0.847)** | **Multilingual**  **BERT**  **(0.926)** | **Thai**  **BERT**  **(0.824)** |
| --- | --- | --- | --- | --- | --- |
| **BioClinical**  **BERT**  **(0.918)** | — | 1.000 | ↑  < 0.001 | 1.000 | ↑  < 0.001 |
| **Clinical**  **BERT**  **(0.927)** | 1.000 | — | ↑  < 0.001 | 1.000 | ↑  < 0.001 |
| **PubMed**  **BERT**  **(0.847)** | ↓  < 0.001 | ↓  < 0.001 | — | ↓  < 0.001 | 1.000 |
| **Multilingual**  **BERT**  **(0.926)** | 1.000 | 1.000 | ↑  < 0.001 | — | ↑  < 0.001 |
| **Thai**  **BERT**  **(0.824)** | ↓  < 0.001 | ↓  < 0.001 | 1.000 | ↓  < 0.001 | — |

**Table S20.** Corrected p-values from Partial F1-score of Time patterns entity comparison among for BioClinicalBERT, ClinicalBERT, PubMedBERT MultilingualBERT, and ThaiBERT on the test datasets.

| **Models**  **(Partial** **F1-score)** | **BioClinical**  **BERT**  **(0.918)** | **Clinical**  **BERT**  **(0.928)** | **PubMed**  **BERT**  **(0.847)** | **Multilingual**  **BERT**  **(0.926)** | **Thai**  **BERT**  **(0.828)** |
| --- | --- | --- | --- | --- | --- |
| **BioClinical**  **BERT**  **(0.918)** | — | 1.000 | ↑  < 0.001 | 1.000 | ↑  < 0.001 |
| **Clinical**  **BERT**  **(0.928)** | 1.000 | — | ↑  < 0.001 | 1.000 | ↑  < 0.001 |
| **PubMed**  **BERT**  **(0.847)** | ↓  < 0.001 | ↓  < 0.001 | — | ↓  < 0.001 | 1.000 |
| **Multilingual**  **BERT**  **(0.926)** | 1.000 | 1.000 | ↑  < 0.001 | — | ↑  < 0.001 |
| **Thai**  **BERT**  **(0.828)** | ↓  < 0.001 | ↓  < 0.001 | 1.000 | ↓  < 0.001 | — |

The ten pairwise (five models) comparisons were performed using two-proportion test with corrected p-value (two-sided) using Bonferroni method. Green with ↑ indicates the row model is statistically better than the column model. Red with ↓ the indicates row model is statistically worse than the column model. Gray indicates no statistically significant difference (p-value ≥0.05 after correction).

**Table S21.** Corrected p-values from Exact F1-score of Unit of presentation entity comparison among for BioClinicalBERT, ClinicalBERT, PubMedBERT MultilingualBERT, and ThaiBERT on the test datasets.

| **Models**  **(Exact** **F1-score)** | **BioClinical**  **BERT**  **(0.889)** | **Clinical**  **BERT**  **(0.892)** | **PubMed**  **BERT**  **(0.889)** | **Multilingual**  **BERT**  **(0.873)** | **Thai**  **BERT**  **(0.398)** |
| --- | --- | --- | --- | --- | --- |
| **BioClinical**  **BERT**  **(0.889)** | — | 1.000 | 1.000 | 1.000 | ↑  < 0.001 |
| **Clinical**  **BERT**  **(0.892)** | 1.000 | — | 1.000 | 1.000 | ↑  < 0.001 |
| **PubMed**  **BERT**  **(0.889)** | 1.000 | 1.000 | — | 1.000 | ↑  < 0.001 |
| **Multilingual**  **BERT**  **(0.873)** | 1.000 | 1.000 | 1.000 | — | ↑  < 0.001 |
| **Thai**  **BERT**  **(0.398)** | ↓  < 0.001 | ↓  < 0.001 | ↓  < 0.001 | ↓  < 0.001 | — |

**Table S22.** Corrected p-values from Partial F1-score of Unit of presentation entity comparison among for BioClinicalBERT, ClinicalBERT, PubMedBERT MultilingualBERT, and ThaiBERT on the test datasets.

| **Models**  **(Partial** **F1-score)** | **BioClinical**  **BERT**  **(0.889)** | **Clinical**  **BERT**  **(0.892)** | **PubMed**  **BERT**  **(0.889)** | **Multilingual**  **BERT**  **(0.876)** | **Thai**  **BERT**  **(0.411)** |
| --- | --- | --- | --- | --- | --- |
| **BioClinical**  **BERT**  **(0.889)** | — | 1.000 | 1.000 | 1.000 | ↑  < 0.001 |
| **Clinical**  **BERT**  **(0.892)** | 1.000 | — | 1.000 | 1.000 | ↑  < 0.001 |
| **PubMed**  **BERT**  **(0.889)** | 1.000 | 1.000 | — | 1.000 | ↑  < 0.001 |
| **Multilingual**  **BERT**  **(0.876)** | 1.000 | 1.000 | 1.000 | — | ↑  < 0.001 |
| **Thai**  **BERT**  **(0.411)** | ↓  < 0.001 | ↓  < 0.001 | ↓  < 0.001 | ↓  < 0.001 | — |

The ten pairwise (five models) comparisons were performed using two-proportion test with corrected p-value (two-sided) using Bonferroni method. Green with ↑ indicates the row model is statistically better than the column model. Red with ↓ the indicates row model is statistically worse than the column model. Gray indicates no statistically significant difference (p-value ≥0.05 after correction).

**Table S23.** Corrected p-values from Exact F1-score of Unit of measure entity comparison among for BioClinicalBERT, ClinicalBERT, PubMedBERT MultilingualBERT, and ThaiBERT on the test datasets.

| **Models**  **(Exact** **F1-score)** | **BioClinical**  **BERT**  **(0.692)** | **Clinical**  **BERT**  **(0.692)** | **PubMed**  **BERT**  **(0.608)** | **Multilingual**  **BERT**  **(0.667)** | **Thai**  **BERT**  **(0.623)** |
| --- | --- | --- | --- | --- | --- |
| **BioClinical**  **BERT**  **(0.692)** | — | 1.000 | 1.000 | 1.000 | 1.000 |
| **Clinical**  **BERT**  **(0.692)** | 1.000 | — | 1.000 | 1.000 | 1.000 |
| **PubMed**  **BERT**  **(0.608)** | 1.000 | 1.000 | — | 1.000 | 1.000 |
| **Multilingual**  **BERT**  **(0.667)** | 1.000 | 1.000 | 1.000 | — | 1.000 |
| **Thai**  **BERT**  **(0.623)** | 1.000 | 1.000 | 1.000 | 1.000 | — |

**Table S24.** Corrected p-values from Partial F1-score of Unit of measure entity comparison among for BioClinicalBERT, ClinicalBERT, PubMedBERT MultilingualBERT, and ThaiBERT on the test datasets.

| **Models**  **(Partial** **F1-score)** | **BioClinical**  **BERT**  **(0.692)** | **Clinical**  **BERT**  **(0.692)** | **PubMed**  **BERT**  **(0.608)** | **Multilingual**  **BERT**  **(0.667)** | **Thai**  **BERT**  **(0.623)** |
| --- | --- | --- | --- | --- | --- |
| **BioClinical**  **BERT**  **(0.692)** | — | 1.000 | 1.000 | 1.000 | 1.000 |
| **Clinical**  **BERT**  **(0.692)** | 1.000 | — | 1.000 | 1.000 | 1.000 |
| **PubMed**  **BERT**  **(0.608)** | 1.000 | 1.000 | — | 1.000 | 1.000 |
| **Multilingual**  **BERT**  **(0.667)** | 1.000 | 1.000 | 1.000 | — | 1.000 |
| **Thai**  **BERT**  **(0.623)** | 1.000 | 1.000 | 1.000 | 1.000 | — |

The ten pairwise (five models) comparisons were performed using two-proportion test with corrected p-value (two-sided) using Bonferroni method. Green with ↑ indicates the row model is statistically better than the column model. Red with ↓ the indicates row model is statistically worse than the column model. Gray indicates no statistically significant difference (p-value ≥0.05 after correction).

**Table S25.** Detials performance metrics on the test datasets

| Models | Entity  types | Exact  Precision | Exact  Recall | Exact  F1 | Partial  Precision | Partial  Recall | Partial  F1 |
| --- | --- | --- | --- | --- | --- | --- | --- |
| **BioClinical**  **BERT** | O | 0.998 | 0.950 | 0.973 | 0.998 | 0.950 | 0.973 |
|  | SUB | 0.970 | 0.860 | 0.911 | 0.973 | 0.862 | 0.914 |
|  | DEC | 0.990 | 0.871 | 0.927 | 0.990 | 0.871 | 0.927 |
|  | ROU | 0.947 | 0.823 | 0.880 | 0.952 | 0.827 | 0.885 |
|  | TIM | 0.979 | 0.865 | 0.918 | 0.979 | 0.865 | 0.918 |
|  | UNI | 0.944 | 0.840 | 0.889 | 0.944 | 0.840 | 0.889 |
|  | MEA | 0.818 | 0.600 | 0.692 | 0.818 | 0.600 | 0.692 |
|  | Overall | 0.996 | 0.942 | 0.968 | 0.997 | 0.942 | 0.969 |
| **Clinical**  **BERT** | O | 0.996 | 0.960 | 0.978 | 0.996 | 0.960 | 0.978 |
|  | SUB | 0.982 | 0.877 | 0.927 | 0.984 | 0.879 | 0.928 |
|  | DEC | 0.992 | 0.882 | 0.934 | 0.992 | 0.882 | 0.934 |
|  | ROU | 0.949 | 0.840 | 0.891 | 0.954 | 0.844 | 0.896 |
|  | TIM | 0.981 | 0.879 | 0.927 | 0.981 | 0.879 | 0.928 |
|  | UNI | 0.965 | 0.830 | 0.892 | 0.965 | 0.830 | 0.892 |
|  | MEA | 0.818 | 0.600 | 0.692 | 0.818 | 0.600 | 0.692 |
|  | Overall | 0.995 | 0.953 | 0.973 | 0.995 | 0.953 | 0.973 |
| **PubMed**  **BERT** | O | 0.995 | 0.877 | 0.932 | 0.995 | 0.877 | 0.932 |
|  | SUB | 0.835 | 0.737 | 0.783 | 0.844 | 0.744 | 0.791 |
|  | DEC | 0.936 | 0.821 | 0.874 | 0.936 | 0.821 | 0.874 |
|  | ROU | 0.932 | 0.807 | 0.865 | 0.937 | 0.811 | 0.870 |
|  | TIM | 0.901 | 0.799 | 0.847 | 0.901 | 0.799 | 0.847 |
|  | UNI | 0.944 | 0.840 | 0.889 | 0.944 | 0.840 | 0.889 |
|  | MEA | 0.738 | 0.517 | 0.608 | 0.738 | 0.517 | 0.608 |
|  | Overall | 0.988 | 0.871 | 0.925 | 0.988 | 0.871 | 0.926 |
| **Multilingual**  **BERT** | O | 0.997 | 0.877 | 0.933 | 0.997 | 0.877 | 0.933 |
|  | SUB | 0.970 | 0.854 | 0.909 | 0.973 | 0.857 | 0.912 |
|  | DEC | 0.994 | 0.873 | 0.930 | 0.994 | 0.873 | 0.930 |
|  | ROU | 0.949 | 0.812 | 0.875 | 0.955 | 0.817 | 0.880 |
|  | TIM | 0.985 | 0.873 | 0.926 | 0.986 | 0.873 | 0.926 |
|  | UNI | 0.927 | 0.825 | 0.873 | 0.930 | 0.828 | 0.876 |
|  | MEA | 0.810 | 0.567 | 0.667 | 0.810 | 0.567 | 0.667 |
|  | Overall | 0.995 | 0.875 | 0.931 | 0.996 | 0.875 | 0.932 |
| **Thai**  **BERT** | O | 0.997 | 0.966 | 0.981 | 0.997 | 0.966 | 0.981 |
|  | SUB | 0.796 | 0.721 | 0.757 | 0.808 | 0.732 | 0.768 |
|  | DEC | 0.944 | 0.829 | 0.883 | 0.945 | 0.830 | 0.884 |
|  | ROU | 0.946 | 0.841 | 0.890 | 0.952 | 0.845 | 0.895 |
|  | TIM | 0.884 | 0.772 | 0.824 | 0.887 | 0.776 | 0.828 |
|  | UNI | 0.458 | 0.353 | 0.398 | 0.472 | 0.364 | 0.411 |
|  | MEA | 0.717 | 0.550 | 0.623 | 0.717 | 0.550 | 0.623 |
|  | Overall | 0.988 | 0.951 | 0.969 | 0.989 | 0.951 | 0.970 |

**Abbreviations:** O, Outside; SUB, Substance; DEC, Decimal; ROU, Route of admisnistration; TIM, Time presentation; UNI, Unit of presentation; MEA, Unit of measure.
